# Synthesis, Characterization, HSA/DNA Binding, and Cytotoxic Activity of [RuCl_2_(η^6^-*p*-cymene)(bph-κ*N*)] Complex

**DOI:** 10.3390/molecules30153088

**Published:** 2025-07-23

**Authors:** Stefan Perendija, Dušan Dimić, Thomas Eichhorn, Aleksandra Rakić, Luciano Saso, Đura Nakarada, Dragoslava Đikić, Teodora Dragojević, Jasmina Dimitrić Marković, Goran N. Kaluđerović

**Affiliations:** 1 Faculty of Physical Chemistry, University of Belgrade, 11000 Belgrade, Serbia; stefan@ffh.bg.ac.rs (S.P.); ddimic@ffh.bg.ac.rs (D.D.); saska@ffh.bg.ac.rs (A.R.); djura@ffh.bg.ac.rs (Đ.N.); 2 Department of Engineering and Natural Sciences, University of Applied Sciences Merseburg, D-06217 Merseburg, Germany; thomas.eichhorn@hs-merseburg.de; 3 Department of Physiology and Pharmacology “Vittorio Erspamer”, Sapienza University of Rome, 00185 Rome, Italy; luciano.saso@uniroma1.it; 4 Institute for Medical Research, University of Belgrade, 11000 Belgrade, Serbia; dragoslava@imi.bg.ac.rs (D.Đ.); teodora.dragojevic@imi.bg.ac.rs (T.D.)

**Keywords:** Ru(II) complex, HSA, DNA, DFT, EPR, molecular docking, in vitro activity

## Abstract

A novel ruthenium(II) complex, [RuCl_2_(η^6^-*p*-cymene)(bph-κ*N*)] (**1**), was synthesized and structurally characterized using FTIR and NMR spectroscopy. Density functional theory (DFT) calculations supported the proposed geometry and allowed for comparative analysis of experimental and theoretical spectroscopic data. The interaction of complex **1** with human serum albumin (HSA) and calf thymus DNA was investigated through fluorescence quenching experiments, revealing spontaneous binding driven primarily by hydrophobic interactions. The thermodynamic parameters indicated mixed quenching mechanisms in both protein and DNA systems. Ethidium bromide displacement assays and molecular docking simulations confirmed DNA intercalation as the dominant binding mode, with a Gibbs free binding energy of −34.1 kJ mol^−1^. Antioxidant activity, assessed by EPR spectroscopy, demonstrated effective scavenging of hydroxyl and ascorbyl radicals. In vitro cytotoxicity assays against A375, MDA-MB-231, MIA PaCa-2, and SW480 cancer cell lines revealed selective activity, with pancreatic and colorectal cells showing the highest sensitivity. QTAIM analysis provided insight into metal–ligand bonding characteristics and intramolecular stabilization. These findings highlight the potential of **1** as a promising candidate for further development as an anticancer agent, particularly against multidrug-resistant tumors.

## 1. Introduction

Multidrug-resistant (MDR) tumors pose a significant challenge in oncology, marked by the ability of cancer cells to resist a wide range of chemotherapeutic agents. This resistance develops through various mechanisms, including the overexpression of efflux transporters such as P-glycoprotein (P-gp), which actively remove drugs from cancer cells, decreasing their intracellular concentrations and effectiveness. Moreover, changes in drug targets, improved DNA repair functions, and the evasion of apoptosis also contribute to the MDR phenotype [1,2,3,4,5].

The development and integration of metallodrugs into cancer therapy hold significant promise for addressing the limitations of current treatments, particularly in combating MDR tumors. By exploiting the unique properties of metal complexes, researchers aim to design more effective and selective anticancer agents that can circumvent resistance mechanisms and improve patient outcomes. These metal-based compounds, such as those containing platinum, ruthenium, copper, and gold, offer unique chemical properties that enable diverse mechanisms of action. Moreover, the structural versatility of metallodrugs enables fine-tuning of their pharmacokinetic and pharmacodynamic profiles, potentially overcoming MDR mechanisms [6,7,8].

For instance, cisplatin, a platinum-based drug, forms DNA crosslinks, in addition to other mechanisms that disrupt replication and transcription, leading to cell death. However, its use is limited by toxicity and resistance issues [8,9,10]. On the other hand, Ru(II) complexes have emerged as promising therapeutic agents in the fight against MDR tumors, offering novel mechanisms of action and improved selectivity compared to traditional chemotherapeutics. Compared to platinum-based drugs, Ru(II) complexes have lower toxicity profiles and can overcome common resistance mechanisms, such as efflux pumps, enhancing their efficacy against MDR tumors. Their chemical properties also enable the selective targeting of cancer cells, thereby sparing healthy tissue. These complexes exhibit multiple anticancer mechanisms, including the induction of apoptosis through mitochondrial dysfunction and the generation of reactive oxygen species (ROS). Ru(II) complexes can also cause DNA damage, leading to cell cycle arrest and apoptosis, often via p53-independent pathways. Certain Ru(II) complexes have been shown to inhibit metastasis by affecting cancer cell adhesion, migration, and invasion, thereby reducing the metastatic potential of tumors. Preclinical studies have shown the effectiveness of Ru(II) complexes in various cancer models, including breast, lung, and colon cancers, positioning them as valuable additions to the anticancer arsenal. Incorporating Ru(II) complexes into therapeutic strategies offers a multifaceted approach to combating MDR tumors, underscoring their importance in the ongoing development of effective cancer treatments [7,8,9,10,11,12,13,14].

This research focuses on a new dichlorido((diphenylmethylidene)hydrazine-κ*N*)(η^6^-*p*-cymene)ruthenium(II) complex ([RuCl_2_(η^6^-*p*-cymene)(bph-κ*N*)]) (**1**). Ruthenium complexes containing ligands with a hydrazine moiety have been described in the literature [15,16,17,18,19,20]. In these papers, the stability of the structure was proven through theoretical calculations, together with significant activity towards free radicals and biomolecules [19,21]. This paper is a continuation of the systematic examination of structural, spectral, and biological activities of Ru(II)-hydrazine complexes by our groups. The binding ability of **1** towards biological molecules such as human serum albumin (HSA) and DNA is supported by fluorescence spectroscopy. The binding interactions of metallodrugs with HSA and DNA are critically important in MDR cancer research. HSA is a key transporter and reservoir for metallodrugs in the bloodstream, influencing their distribution, stability, and bioavailability. By binding to HSA, metallodrugs can achieve prolonged circulation times, enhanced delivery to tumor sites, and reduced systemic toxicity. Once in the tumor microenvironment, the ability of metallodrugs to interact directly with DNA is fundamental to their anticancer activity, as they often induce DNA damage to trigger cell death. In MDR cancers, where resistance mechanisms such as drug efflux pumps limit the effectiveness of conventional chemotherapeutics, the dual interaction of metallodrugs with HSA and DNA offers promising strategies to improve drug delivery and overcome resistance. Efficient HSA binding can facilitate targeted delivery, while strong DNA associations can enhance cytotoxic effects even in resistant cancer cells, making these interactions a focal point in developing more effective metallic anticancer agents [22,23,24]. Furthermore, molecular docking analysis is performed to obtain detailed information about the binding interaction modes of the complexes with DNA and HSA. Generally, molecular docking of HSA and DNA with metallodrugs is of great importance in MDR cancer research as it allows for the prediction and analysis of how metallodrugs interact with these biological targets at the molecular level, providing insights into binding affinity, interaction sites, and binding modes [24,25]. Assessing the cytotoxicity of **1** is performed by employing the in vitro MTT test which provides insights into the metabolic activity of living cells enabling evaluation of cytotoxic effects of **1** on MDR cancer cells like A375 (malignant melanoma), MDA-MB-231 (breast adenocarcinoma), MIA PaCa-2 (pancreatic carcinoma), and SW480 (colorectal adenocarcinoma), all used in this study [26].

## 2. Results and Discussion

### 2.1. Synthetic Procedure

**Scheme 1:**
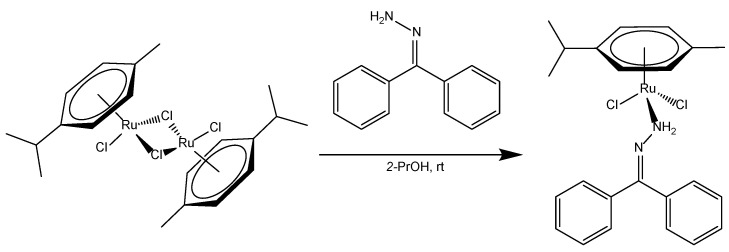
Synthesis of [RuCl_2_(η^6^-*p*-cymene)(bph-κ*N*)].

The complex was prepared in 2-propanol at room temperature in the mixture of [{RuCl_2_(η^6^-*p*-cymene)}_2_] and (diphenylmethylidene)hydrazine. The reaction occurred under an inert nitrogen atmosphere, according to Scheme 1. The formation of an orange suspension was observed. The reaction mixture was left overnight at room temperature, after which diethyl ether was added to the suspension. The resulting suspension was filtered, washed with diethyl ether, and air-dried. The reaction yield was 45.7%. The presence of **1** was supported by FTIR and NMR spectroscopy. Purity was confirmed with elemental analysis. These spectra are assigned and discussed in more detail in the following section.

### 2.2. Optimization of Structure

**Figure 1:**
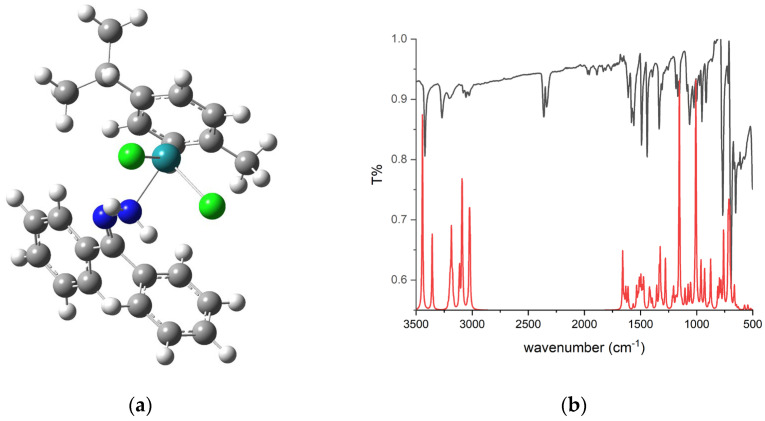
(**a**) Optimized structure at the B3LYP/6-311++G(d,p)(H,C,N,Cl)/def2-TZVP(Ru) level of theory and (**b**) experimental (black line) and theoretical (red line) FTIR spectra of [RuCl_2_(η^6^-*p*-cymene)(bph-κ*N*)].

As the crystallization attempts failed, theoretical chemistry methods were used for the structural elucidation of 1. The predicted structure was prepared based on the analogous compounds [19,20,21], as similar structural elements were present. The level of theory chosen for the optimization of the structure was B3LYP/6-311++G(d,p)(H,C,N,Cl)/def2-TZVP(Ru), as it was shown that the predicted structural parameters of similar compounds were comparable to the crystallographic ones, as in [20]. The optimized structure is presented in Figure 1.

The structure of **1** consists of two chlorido ligands, a *p*-cymene moiety, and a (diphenylmethylidene)hydrazine. The bond distances between the chlorido ligands and the central metal ion are 2.444 and 2.454 Å. The same bond lengths were previously found in the optimized structures of Ru(II) complexes containing *m*-nitrophenylhydrazine and *m*-chlorophenylhydrazine ligands [21]. Also, the crystallographic structures of similar compounds in [19,20] have Ru−Cl bond lengths between 2.4007(15) and 2.4481(14) Å. This distance range for **1** falls within the expected values of such complexes [19]. The bond distance Ru−N is 2.190 Å, almost the same as in the previously mentioned crystallographic and optimized structures. This result verifies that the choice of ligand does not influence the bond distance between Ru(II) and the hydrazine ligand. This is a consequence of the presence of the N−N bond that prevents the extended delocalization of this part of the molecule. The pseudo-octahedral geometry is preserved, as three metal-ligand interactions are formed with the *p*-cymene moiety. The average Ru−C bond distance is 2.253 Å. In the crystallographic structures of similar compound with a hexamethylbenzene moiety, the bond lengths were comparable [27]. The experimental bond lengths are usually shorter than the theoretical ones, as there is a relaxation due to optimizing an isolated compound in vacuum. The bond angle between chlorido ligands and Ru(II) is 89.41°, while in optimized analogous structures with *m*-nitrophenylhydrazine and *m*-chlorophenylhydrazine ligands, the values were 90.09 and 90.32°, respectively [22]. In the case of a complex with 3,4-dimethylphenylhydrazine, the Cl−Ru−Cl value was 88.92° [20] The angle N–Ru-Cl is usually prone to change due to the interactions between ligands. In the present structure, the angles N−Ru−Cl are 80.07 and 80.18°. These values are lower than in complex 3,4-dimethylphenylhydrazine because of the bulkiness of (diphenylmethylidene)hydrazine.

### 2.3. Comparison of Experimental and Theoretical FTIR Spectra

The experimental and theoretical FTIR spectra of **1** are presented in Figure 1b. The theoretical spectrum of the complex was calculated, and the peak positions and intensities are shown without any correction. The most intense peak at 3415 cm^−1^ in the region above 3000 cm^−1^ in the experimental spectrum is attributed to the asymmetric N−H stretching vibration of the hydrazine bph ligand. The symmetric vibration of the same group is positioned at lower frequencies, namely at 3286 cm^−1^ in the experimental and 3363 cm^−1^ in the theoretical spectrum. The low-intensity peak at 3200 cm^−1^ in the experimental spectrum is assigned to the aromatic C−H stretching vibration of *p*-cymene and hydrazine moieties. The same peak is located at 3188 cm^−1^ in the theoretical spectrum and consists of several transitions. The aliphatic C−H vibrations are assigned to a broad peak between 3078 and 3012 cm^−1^. There are two intense peaks at 3098 and 3020 cm^−1^ in the theoretical spectrum due to asymmetric and symmetric vibrations of methyl groups of the *p*-cymene moiety. It should be noted that the wavenumbers of polar groups typically depend on the physical state of the sample, as intermolecular interactions stabilize the system. In this case, the central metal ion is surrounded by ligands that lack polar groups that readily interact with surrounding molecules in the solid phase, resulting in comparable wavenumber values. A doublet near 2400 cm^−1^ is attributed to atmospheric carbon dioxide.

In the region below 1660 cm^−1^, several peaks are important for the structural characterization of the compound. The peak assigned to C=N stretching vibration is positioned at 1656 cm^−1^ in the theoretical and 1611 cm^−1^ in the experimental spectrum. Several peaks between 1585 and 1546 cm^−1^ are attributed to the combination of C=C and C=N vibrations. There are two medium-intensity peaks at 1488 and 1437 cm^−1^ in the experimental spectrum. The theoretical analysis allows their identification as peaks due to C=C vibration (1474 cm^−1^) and bending C−H (1420 cm^−1^) vibrations. Other medium intensity peaks at 1333 and 1324 cm^−1^ in experimental and theoretical spectra are assigned to the in-plane bending vibration of the amino group, which is consistent with previous research [21]. The rocking vibration of the N−H group is assigned to peaks 1154 and 1171 cm^−1^ in experimental and theoretical spectra, respectively. Slightly lower wavenumbers of the amino group vibrations result from the interaction between the central metal ion and nitrogen atom, leading to the respective bonds’ elongation. The stretching N−N vibration is located at 1000 and 1048 cm^−1^ in experimental and theoretical spectra, identified based on the spectrum of hydrazine and visualization of vibrational motion [28]. The twisting N−H vibration is visible in both spectra as an intense peak at 760 cm^−1^. The Ru−N stretching vibration is present in the range below 500 cm^−1^; therefore, it is not visible in the experimental spectrum but at 439 cm^−1^ in the theoretical spectrum. The theoretical analysis allows the identification of Ru−Cl vibrations at 286 and 269 cm^−1^. Similar wavenumbers were previously determined for the trichloridotriaquoruthenium(III) complex [29]. The position of the Ru−Cl band can be used to classify the bridging and terminal ligands [30]. Based on the analysis and comparison of FTIR spectra, it can be concluded that the optimized structure accurately represents the experimental one.

### 2.4. Comparison of Experimental and Theoretical NMR Spectra

The experimental NMR spectra were recorded in DMSO. On the other hand, the theoretical spectra were calculated for the structure of complex **1**, optimized in DMSO as the solvent, to include the effects of the solvent on the chemical shifts. Table S1 shows the experimental and calculated ^1^H and ^13^C NMR spectra of **1**. The comparison of the two data sets was performed using the correlation coefficient (R) and the mean absolute error (MAE). The MAE indicates the average absolute difference between the observed experimental chemical shifts and the predicted theoretical values. The calculated values were overestimated because the spectra prediction was performed for an isolated molecule in a polarizable continuum, without considering specific solvent-solute interactions.

In the ^1^H NMR spectrum, the lowest chemical shift values, as expected, were obtained for the methyl protons of the isopropyl moiety (1.15 ppm in the experimental and 1.33 ppm in the theoretical spectrum). The methyl protons from the *p*-cymene moiety resonate at 2.04 (exp.) and 2.01 (theor.) ppm. The proton of the CH group in the isopropyl moiety has a chemical shift of 2.79 ppm, with characteristic septet, in the experimental spectrum and 2.91 ppm in the theoretical spectrum. The protons of *p*-cymene form a multiplet centered around 5.75 ppm in the experimental (4.66 ppm in the theoretical) spectrum. The signals assigned to protons in the structure of (diphenylmethylidene)hydrazine are between 7.23 and 7.48 ppm in the experimental and between 7.30 and 8.05 ppm in the theoretical spectrum. The correlation coefficient for predicted and experimental data sets is 0.992, while the MAE value is 0.31 ppm.

The correlation coefficient for ^13^C NMR chemical shifts is 0.997, with an MAE value of 3.4 ppm. The signals of carbon atoms are usually less prone to the effects of solvents and other present chemical species. The signals at 18.55 and 19.48 ppm in experimental and theoretical spectra are assigned to the methyl group carbon atoms of the isopropyl moiety. Slightly higher values were found for the third methyl group at 22.15 (22.56) ppm. The carbon atoms of the *p*-cymene moiety resonate at 86.17, 87.00, 100.77, and 107.07 ppm in the experimental (theoretical: 81.89, 82.21, 99.07, and 105.29) spectrum. Several resonances were assigned to the carbon atoms of the bph ligand, between 126.12 and 145.37 ppm. These results additionally prove the applicability of the selected level of theory for the optimization of the structure that is used to examine the stabilization interactions and later for the molecular docking simulations.

### 2.5. QTAIM Analysis of Intramolecular Interactions

**Figure 2:**
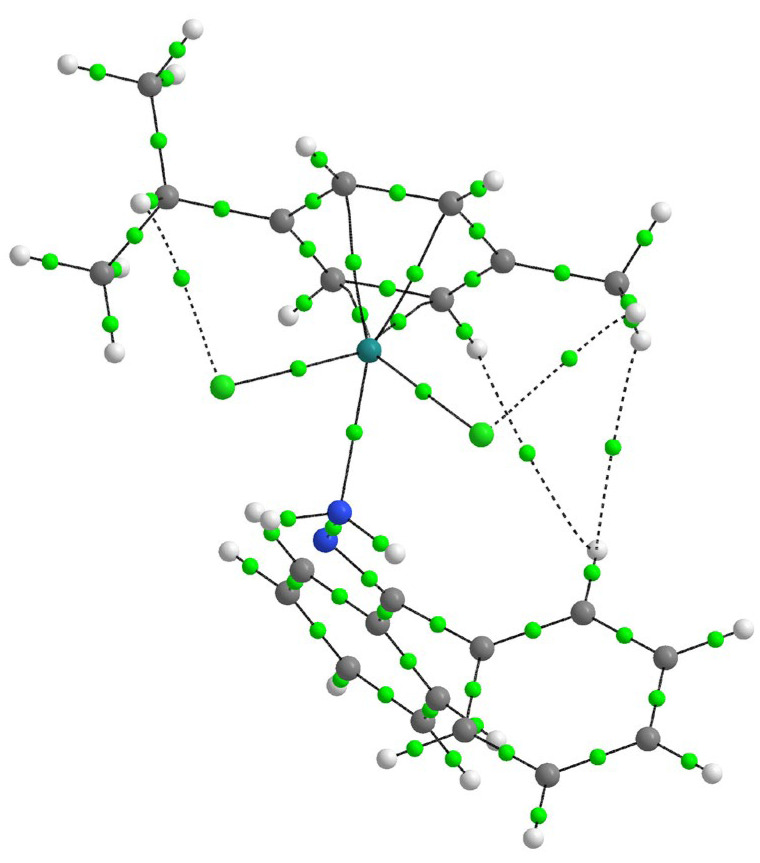
Bond Critical Points (BCPs, green dots) in the structure of **1** (optimized at B3LYP/6-311++G(d,p)(H,C,N,Cl)/def2-TZVP(Ru)).

The coordination environment and strength of metal-ligand interactions in ruthenium complexes were analyzed through the Quantum Theory of Atoms in Molecules (QTAIM), a method recognized for quantifying bonding interactions and characterizing electron density distributions [31,32,33]. For selected bonds, the analysis involved evaluating several topological parameters at the Bond Critical Points (BCPs), including electron density (ρ(r)), its Laplacian (∇^2^ρ(r)), Lagrangian kinetic energy density (G(r)), potential energy density (V(r)), total energy density (H(r) = G(r) + V(r)), and interatomic bond energy (E_bond_ = V(r)/2) [34]. The interaction energies were determined using the potential energy density according to the methodology proposed by Espinosa [35]. The classification of bond types was further refined by Bianchi et al., who proposed using the ratio −G(r)/V(r) as a diagnostic parameter. According to their criteria, covalent interactions are associated with values below 1, ionic interactions with values above 2, while those in the range of 1 to 2 are indicative of intermediate, partially covalent bonding [36]. Electron density values at BCPs serve as a primary criterion for interaction classification. Bader and Essen suggested that values exceeding 0.1 a.u. indicate shared (covalent) interactions, whereas lower values, typically around 0.01 a.u., are characteristic of closed-shell interactions such as hydrogen bonding, ionic, or van der Waals forces [37,38]. Additionally, the total electron energy density (H(r)) sign offers insight into the bonding character—negative values generally imply a covalent nature. Table 1 presents the calculated QTAIM parameters for **1**. Only the most important ligand-central metal ion interactions are selected, along with weak interactions that stabilize the overall structure of the complex. BCPs of **1** are visualized in Figure 2.

The interactions listed in Table 1 reflect the previously discussed structure of **1**. The interactions between carbon atoms of the *p*-cymene moiety and Ru(II) are characterized by the electron densities in a very narrow range (0.082–0.086 a.u.), and Laplacian values around 0.240 a.u. These interactions have a partial covalent character with a negative total electron energy value, between −51.1 and −58.1 kJ mol^−1^, and a −G(r)/V(r) value of 0.8. The interaction energies are around −130 kJ mol^−1^, and the actual values depend on the position of the carbon atoms. However, it can be expected that these energies will be equilibrated due to the symmetry of the solution. The interactions between chlorido ligands and the central metal ion are weaker than those with carbon atoms, with lower electron density (0.086 a.u.) and Laplacian (0.180 a.u.) values. The total electron energy is negative while maintaining a partial covalent character. The interaction energies are also lower (−94.4 and −92.1 kJ mol^−1^). The Ru−N interaction has an electron density value of 0.077 a.u. and a Laplacian of 0.293 a.u. The partial covalent character is shown by the H(r) value of −33.9 and the −G(r)/V(r) value of 0.9, similar to other Ru−N bonds in organometallic dyes [32]. The interaction strength is comparable to that of carbon atoms.

The optimized structure is additionally stabilized by the presence of much weaker interactions between chlorido ligands and hydrogen atoms of the *p*-cymene moiety. These interactions have much lower electron density (0.008 a.u.) and a −G(r)/V(r) value of 1.3. The interaction energies are −5.6 and −5.2 kJ mol^−1^. Another type of interaction is denoted as H∙∙∙H with energies of only −0.9 kJ mol^−1^.

### 2.6. Antioxidant Activity

**Figure 3:**
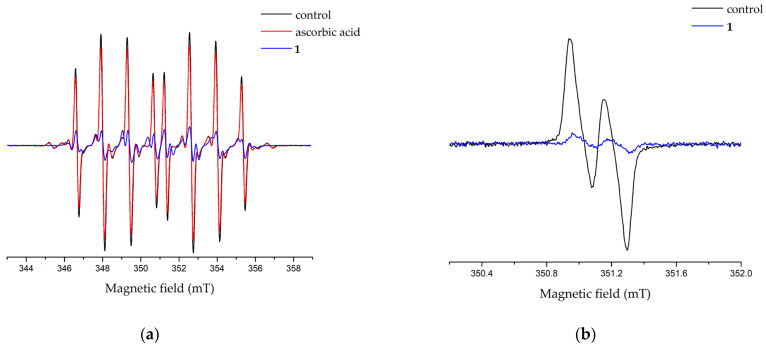
The experimental EPR spectra of the (**a**) DEPMPO-HO^•^ adduct (black line), ascorbic acid (red line) and **1** (blue line), and (**b**) ascorbyl radical without (black line) and with added **1** (blue line).

The antioxidant activity of **1** was examined towards two radicals, HO^•^ and ^•^Asc, by EPR spectroscopy. These two radicals are representatives of biologically relevant radicals with different half-lives. The first radical is short-lived, and the spin trap technique is necessary for its detection. The second radical has a characteristic EPR spectrum, and its amount can be directly monitored. The HO^•^ was generated in the Fenton system, and a DEPMPO spin trap was used. Figure 3 presents EPR spectra of DEPMPO-HO^•^ species without and with added **1** and ascorbic acid, as a reference compound. The amount of radicals was calculated from the intensities of the peaks, as shown in the methodology section. Upon the addition of **1**, weak additional peaks appeared in the spectrum, attributed to the presence of DMSO solvent. However, these peaks did not significantly overlap with the characteristic peaks of DEPMPO-HO^•^ adduct, so they did not interfere with its detection. The same final concentrations of ascorbic acid and **1** (16 µM) were used for the radical scavenging. The amount of reduced radical was 84.6% in the case of **1** and 11.0% for the reference compound. These results show that **1** is a better antioxidant than ascorbic acid. The structural features of **1** somewhat limit the possible radical scavenging mechanism, as no groups readily donate protons. Therefore, it can be discussed that radical adduct formation is a probable reduction mechanism [39].

The EPR spectrum of ascorbyl radical is much simpler, and the concentration of the present radical was also monitored through the intensity of the peaks. The amount of reduced radical is 90.1%, proving that **1** can reduce even more stable radicals than previously discussed. These results also show that the newly obtained complex can be used to protect cells from oxidative stress and reactive species.

### 2.7. Experimental Assessment of Protein Binding Affinity

Due to its high binding capacity and conformational flexibility, HSA is widely used as a model protein for studying ligand interactions. Its intrinsic fluorescence primarily originates from a single tryptophan residue (Trp214) located in subdomain IIA, which is highly sensitive to changes in its local environment [40]. This characteristic makes tryptophan fluorescence a valuable tool for investigating protein-ligand interactions, as the binding of small molecules or metal complexes near this residue can lead to changes in fluorescence intensity or shifts in emission wavelength due to quenching effects.

Fluorescence quenching can occur through different mechanisms, including static, dynamic, or a combination of both. The relationship between the Stern-Volmer constant (K_SV_) and temperature provides insights into the quenching mechanism: an increase in K_SV_ with rising temperature indicates dynamic quenching, which involves collisions and diffusion of quencher molecules, while a decrease suggests static quenching, where complex formation occurs between the quencher and HSA. For calculations, the intercept in the Stern-Volmer plot was fixed at one to simplify interpretation [20,40,41,42].

**Figure 4:**
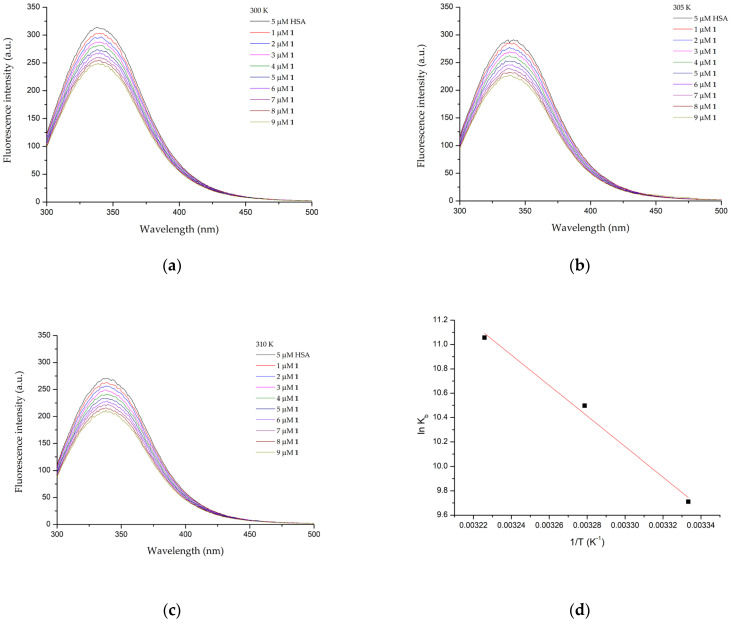
The fluorescence spectra of HSA after the addition of various concentrations of **1** at (**a**) 27 °C; (**b**) 32 °C; (**c**) 37 °C; and (**d**) Van’t Hoff diagram for the binding process of **1** and HSA.

In the experimental setup, HSA solutions were excited at 285 nm, a wavelength that efficiently excites tryptophan and tyrosine residues, allowing the monitoring of fluorescence changes upon ligand binding. Fluorescence emission spectra were recorded at varying concentrations of the complex and at three temperatures. Figure 4 presents the fluorescence spectra of HSA after adding various concentrations of **1**. Table 2 lists all relevant parameters obtained from the experimental spectra.

Upon adding the **1**, the intensity of HSA fluorescence emission significantly lowered. The SV quenching constants were between 2.85 × 10^4^ and 3.23 × 10^4^ M^−1^ in the 300–310 K temperature range. As the temperature increases, the constant values also rise, which indicates that the quenching of HSA fluorescence occurs through a dynamic mechanism.

The binding constants were 1.65 × 10^4^ (300 K), 3.62 × 10^4^ (305 K), and 6.34 × 10^4^ M^−1^ (310 K). For the same set of values, the parameter n was between 0.95 and 1.06, proving that one molecule of **1** is attached to one molecule of HSA. The measurements at three temperatures allowed the determination of the thermodynamic parameters of binding.

The enthalpy change for binding was determined to be 104.149 kJ mol^−1^, while the entropy change was 482.179 J mol^−1^ K^−1^ (Table 2). The Gibbs free energy change ranged from −24.3 to −28.6 kJ mol^−1^, indicating that the binding process is spontaneous. Based on the thermodynamic parameters and their influence on the Gibbs free energy, it can be concluded that hydrophobic interactions primarily drive the binding process. The nature of the ligand and the complex in general indicate the presence of van der Waals interactions with HSA, as determined by the positive changes in enthalpy and entropy.

### 2.8. Experimental Assessment of DNA Binding Affinity

#### 2.8.1. Spectrofluorimetric Titration of **1** by CT-DNA

Elucidating the interaction between the complex and DNA serves as the first step in biological evaluation, leading to anticancer activity and application in tumor therapy [43,44]. Calf thymus DNA (CT-DNA) is commonly used as a model system for investigating DNA-binding properties of small molecules and metal complexes. Studying such interactions is essential for understanding potential mechanisms of action for DNA-targeted therapeutics and evaluating binding modes such as intercalation, groove binding, or electrostatic interactions. Although native DNA is only weakly fluorescent, its interactions with fluorescent molecules or metal complexes can be monitored via spectrofluorimetric titration. When a metal complex binds to DNA, it can lead to changes in the fluorescence intensity of either the complex or a DNA-bound fluorescent probe. These changes provide valuable information about binding affinity, stoichiometry, and mode of interaction. CT-DNA is especially useful in these studies due to its structural similarity to human DNA and well-characterized base composition.

Complex showed stable fluorescence maximum at 329 nm upon irradiation at 217 nm. The electronic emission spectra were recorded between 237 and 400 nm upon the addition of CT-DNA, as explained in the reference [41]. Measurements were repeated at three temperatures (Figure S2). Table 3 displays the KSV values, binding constants, the number of binding sites (Hill coefficient), and the change in Gibbs free energy of binding.

The trend of the Stern–Volmer constant values shows a slow increase (1.86–1.98 × 10^4^ M^−1^) with rising temperature, indicating that the fluorescence quenching is dynamic, occurring through space via dipole–dipole interactions. The binding constant values also increase with temperature, although the differences between values are more pronounced. The values of change in enthalpy and entropy of binding are positive, 124.635 kJ mol^−1^ and 487.683 J mol^−1^ K^−1^. The values of the change in Gibbs free energy are −21.7, −24.1, and −26.5 kJ mol^−1^ for 300, 305, and 310 K, respectively. The fluorescence quenching experiments of 1 with CT-DNA indicate that the binding process is spontaneous. The actual binding mechanism at the molecular level is examined in the molecular docking simulation section.

#### 2.8.2. Ethidium Bromide Displacement Studies

Ethidium bromide (EB) displacement studies are used to investigate how compounds interact with DNA by examining their ability to displace EB from its intercalated position within the DNA helix [44]. By intercalating into DNA, EB forms a highly fluorescent compound (CT-DNA–EB) with excitation at 520 nm and emission at 600 nm [45,46]. When the tested compound is added, CT-DNA-EB dissociates, resulting in a reduction in fluorescence intensity. Unbound EB is quickly quenched by species present in the solution. This method helps determine the binding mode and strength of the compound’s interaction with DNA, providing insight into whether the binding occurs through intercalation or groove binding.

Figure S3 shows the emission spectra of CT-DNA–EB in the presence of different concentrations of 1 at 27 °C. As the concentration of 1 increases, the fluorescence intensity diminishes, as expected. The calculated Stern–Volmer quenching constant (K_sv_) is 7.87 × 10^3^ M^−1^, the binding constant calculated using the double-log Stern–Volmer equation is 1.70 × 10^6^ M^−1^, and the coefficient (n) is determined to be 1.5. These findings suggest that approximately one EB molecule is displaced per complex molecule, although the displacement can occur through either intercalative or groove-binding modes, depending on the size and shape of the complex, as explored in molecular docking studies. The estimated binding free energy, calculated from the binding constant, is −35.8 kJ mol^−1^.

### 2.9. Molecular Docking Simulation of HSA/DNA Binding

**Figure 5:**
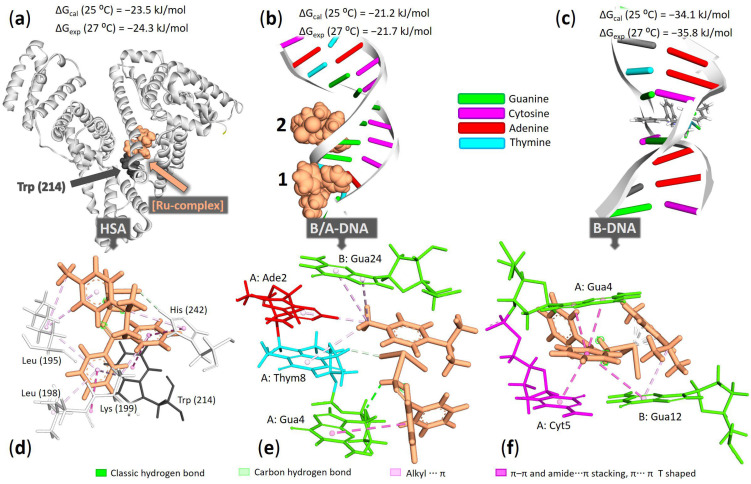
Adducts between the **1** and selected biomolecular targets: (**a**) HSA, (**b**) intermediate structure between B- and A-form DNA (B/A-DNA), and (**c**) canonical B-DNA. The calculated change in Gibbs free energy of binding (∆G_cal_) and the experimentally determined change in Gibbs free energy (∆G_exp_) are shown above each figure. Complexes are depicted in orange using a ball representation for overall visualization. For clarity, only the intercalated **1** is shown in stick representation, colored by atom type. Detailed orientations of the **1** with the best ∆G_cal_ values represented as orange sticks together with their intermolecular interactions are displayed in the following binding environments: (**d**) FA7 site on HSA, (**e**) minor groove of DNA with the most favorable ∆G_cal_ (site 1), and (**f**) intercalation site within B-DNA. All intermolecular interactions between the **1** and the biomolecules are listed in Table S3.

The molecular docking simulations were conducted to complement and rationalize the experimental findings obtained from spectrofluorimetric measurements, including displacement of ethidium bromide from DNA, fluorescence quenching of HSA, and spectrofluorimetric titration of **1** with DNA. The results of the docking studies are summarized in Table 4, while the predicted binding modes of **1** with various biomolecular targets are illustrated in Figure 5.

In detail, Figure 5a depicts the binding of compound **1** within the FA7 binding pocket of HSA, located above the Trp214 residue. Figure 5b shows the interaction of **1** with the A/B intermediate form of DNA, and Figure 5c illustrates its intercalation into the DNA double helix. The lower panels (Figure 5d–f) provide magnified representations of the specific intermolecular interactions established between **1** and its respective biomolecular targets. A notable consistency was observed between the experimentally determined Gibbs free energy changes (∆G_exp_, at 27 °C) and the binding free energies predicted by molecular docking (∆G_calc_, at 25 °C), indicating that the docking simulations are in good agreement with spectrofluorimetric data. Structural distinctions among major DNA conformations are discussed in the Supplementary Materials (Table S2).

Compound **1** is composed of three aromatic rings (Figure 1a), suggesting a high capacity for engaging in π∙∙∙π stacking interactions. Based on its structural features, it can be anticipated that aromatic environments, such as nucleobases in DNA and aromatic amino acid residues like tryptophan, phenylalanine, and tyrosine in proteins, would enhance its binding affinity through π∙∙∙π interactions.

Accordingly, stronger binding energies are expected to correlate with a greater number of π∙∙∙π stacking interactions. Experimentally, the most favorable binding assembly was observed for the intercalation of **1** into DNA (–35.8 kJ mol^−1^), followed by binding to HSA (–24.3 kJ mol^−1^), and interaction with the DNA double-strand (–21.7 kJ mol^−1^). For clarity and coherence, the docking results are presented in descending order of binding affinity and are interpreted in the context of the number and nature of π∙∙∙π stacking interactions involved.

The most favorable binding site for the investigated complex was identified within the intercalation region of DNA, where the aromatic nitrogenous bases are arranged in a parallel fashion above and below the inserted complex (Figure 5c). One benzene ring of the (diphenyl-methylidene) hydrazine moiety inserts in the layer between two adjacent base pairs without traversing to the opposite DNA strand or interacting with the sugar-phosphate backbone. This partial insertion results in four π∙∙∙π stacking interactions (Figure 5f), involving the aromatic rings of Guanine 4 and Cytosine 5 (chain A), as well as Guanine 12 (chain B). The second benzene ring and the *p*-cymene moiety remain outside the intercalation site, oriented toward the minor groove, indicative of a semi-intercalative [47] binding mode from the minor groove side.

The three-dimensional architecture of compound 1, supported by the flexibility of its alkyl spacer, allows for conformational adjustment within the spatially constrained intercalation cavity of DNA. Its specific geometry, characterized by the spatial orientation of three aromatic rings along distinct axes, permits the insertion of only one benzene ring into the intercalation site. The ancillary aromatic rings within the DNA minor groove are laterally positioned relative to the intercalating benzene ring. The inability of the remaining aromatic rings to align coaxially with the intercalating one prevents full insertion between adjacent base pairs. As a result, **1** cannot bridge the intercalation site entirely, unlike classical intercalators such as ethidium bromide, which span from the minor to the major groove, fully embedding between the stacked base pairs. Intercalation of aromatic ligands typically occurs in a sequence-independent manner, although some preference for GC-rich regions has been reported. Rigid, fused aromatic systems are structurally well-suited for classical intercalation, whereas ligands containing shorter or non-fused aromatic moieties often fail to insert between adjacent base pairs fully. In such cases, auxiliary ligand components may instead form interactions with the sugar-phosphate backbone or occupy the major or minor grooves, thereby restricting complete intercalation [48].

In the present study, the intercalating benzene ring of **1** is positioned at the center of the intercalation cavity. Below and above, its π-system is vertically aligned with the two inter-base-pair spaces. Each of the spaces is flanked by laterally displaced aromatic bases of the DNA duplex. This arrangement favors energetically advantageous displaced π∙∙∙π stacking interactions. Moreover, the spatial configuration places the partially positive peripheral hydrogens of the surrounding DNA bases directly above and below the electron-rich π-cloud of the intercalating benzene ring in **1**, further stabilizing the interaction. Notably, the calculated binding free energy for this mode of interaction is −34.1 kJ mol^−1^, which significantly exceeds the corresponding value for ethidium bromide (–19.5 kJ mol^−1^). These results indicate a potentially stronger binding affinity of **1** and suggest its capability to displace conventional intercalators competitively.

When interactions with HSA are concerned, the most probable interaction site is FA7 (Figure 5a), with the best value of a calculated change in Gibbs free energy of binding of −23.5 kJ mol^−1^. Although the **1** forms the greatest number and diversity of interactions at this site, including five π∙∙∙alkyl interactions with Lys195, Leu198, and Lys199, the overall binding energy is lower than that observed for the DNA intercalation site (−34.1 kJ mol^−1^). Additional interactions include a parallel-displaced π∙∙∙π stacking interaction with the imidazole ring of His242 and a T-shaped π∙∙∙π interaction with Trp214. One benzene ring also interacts with the amide bond between Leu198 and Lys199. Moreover, a carbon-hydrogen bond is formed between the chlorine atom coordinated to Ru(II) and the C–H group of His242’s imidazole ring. The methyl and isopropyl groups of *p*-cymene establish alkyl–alkyl (with Lys195) and alkyl∙∙∙π interactions (with His242), respectively. After all iterations, **1** consistently binds to the FA7 site near Trp214, engaging residues from the IIA subdomain. The most energetically favorable binding pose of the **1** (ΔG = −23.5 kJ mol^−1^) reveals direct interaction with the Trp214 residue, which strongly suggests a static fluorescence quenching mechanism through the formation of a non-emissive ground-state complex. In contrast, an alternative binding configuration, with a comparable binding free energy (ΔG = −22.7 kJ mol^−1^), positions the **1** farther from Trp214. In this pose, no direct contact with the intrinsic fluorophore is established; instead, only weak π∙∙∙alkyl interactions are observed with amino acid residues Arg218, Leu219, Leu238, and Ala291. This spatial arrangement is more consistent with dynamic quenching, which occurs via collisional encounters between the fluorophore and the quencher during the excited state.

The small difference in calculated binding free energy (ΔG = 0.8 kJ mol^−1^) between the two poses suggests that both orientations are thermodynamically accessible under physiological conditions, thereby supporting the possibility of a mixed quenching mechanism. While molecular docking results slightly favor static quenching through complexation at the Trp214 site, experimental Stern–Volmer fluorescence analysis supports a dynamic quenching process. These observations are not mutually exclusive; rather, they are complementary to one another. They can be attributed to the high conformational flexibility of HSA in aqueous solution, which allows for both transient and more stable fluorophore–quencher interactions. Thus, it is reasonable to conclude that both static and dynamic contributions are likely involved in the observed fluorescence quenching behavior of the **1** in the presence of HSA.

The change in Gibbs free energy derived from the spectrofluorimetric titration of **1** with DNA was found to be insufficiently negative to support classical intercalation [49]. Although the docking results presented in Table 4 indicate that intercalation is energetically favorable, it is likely hindered by kinetic barriers. Consequently, alternative binding modes such as minor groove binding or electrostatic interactions with the sugar-phosphate backbone are considered more probable [47]. Another important factor is the known selectivity of certain ruthenium complexes for specific DNA conformations. While some complexes exhibit preferential binding to B-form DNA, others favor A-form DNA structures. In this study, efforts were made to explore a comprehensive range of DNA conformations available in the Protein Data Bank (PDB) in order to determine which structural form yields the best agreement with experimental findings.

Among the analyzed DNA structures, the calculated Gibbs free binding energies were as follows: A-DNA (–23.0 kJ mol^−1^), A/B-DNA (–21.2 kJ mol^−1^), B-DNA (–25.1 kJ mol^−1^), and Z-DNA (–26.8 kJ mol^−1^). Interestingly, the A/B intermediate form showed the closest match to the experimentally determined value (–21.7 kJ mol^−1^), suggesting that of 1 binding may induce or stabilize an A/B-type DNA conformation. The optimal binding site was located in the minor groove, involving interactions exclusively with chain A. Key contacts included hydrophobic interactions between the methyl group of *p*-cymene and the pyrimidine rings of Adenine 2, Thymine 3, and Guanine 24. Additionally, the amino group and the coordinated chlorine of **1** formed hydrogen bonds with the sugar ring of Guanine 4 and the pyrimidine ring of Thymine 3. A single T-shaped π∙∙∙π interaction was also observed between one benzene ring of **1** and the pyrimidine moiety of Guanine 4. It should be noted that alternative binding modes might be present if DNA dynamics is taken into consideration.

Muralisankar et al. investigated the binding of eight ruthenium(II)-arene complexes with triarylamine-thiosemicarbazone hybrids to DNA using spectrophotometric titration. The binding constant was determined to be in the range 2.39–3.03 × 10^4^ M^−1^. It is also reported binding constants to HSA from spectrofluorimetric titrations, and these constants lie in the range 2.75 × 10^4^ M^−1^ to 1.39 × 10^4^ M^−1^ [50].

In a separate study, Đukić el al. showed that their ruthenium(II)-arene complexes with different substituted isothiazole ligands bind strongly to HSA with binding constant which for one complex (4) reaches 1.04 × 10^6^ M^−1^ with a corresponding Gibbs free energy of −34.3 kJ/mol. For the same complex (4), the interaction with CT-DNA was examined with a binding constant of 4.97 × 10^4^ M^−1^ and ΔG° of −26.8 kJ mol^−1^ [2].

Finally, Patel and coworkers examined the interaction of Ru(II)-acylthiourea complexes with both HSA and DNA. Binding constants and Gibbs free energy were observed for both biomacromolecules, and for example for complex 6 they are around 0.7 × 10^5^ M^−1^**,** with ΔG° value close to −27.8 kJ/mol for HSA interactions and 5.8 × 10^3^ M^−1^ for DNA interactions. These findings suggest a comparable binding affinity and possibly a similar mechanism of interaction, likely driven by hydrogen bonding and hydrophobic effects [51].

### 2.10. In Vitro Activity

#### 2.10.1. MTT Test

**Figure 6:**
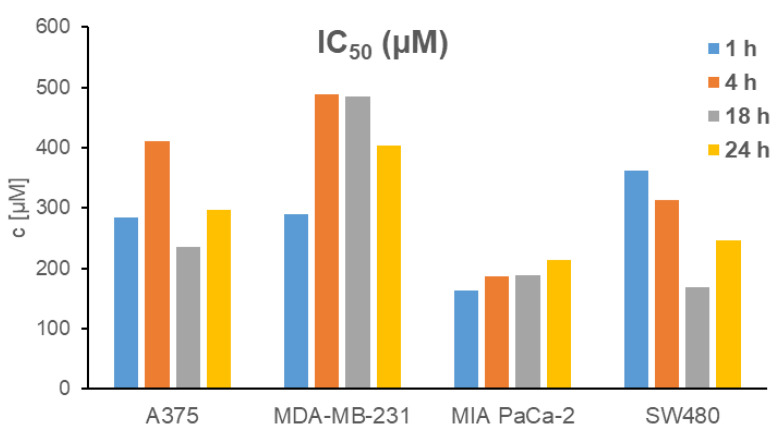
IC_50_ values for treating A375, MDA-MB-231, MIA PaCa-2, and SW480 cells for 1, 4, 8, and 24 h by complex **1**.

The cytotoxicity of **1** was tested against A375, MDA-MB-231, MIA PaCa-2, and SW480 cancer cell lines. Before the experiment, the stability of complex 1 was monitored in DMSO for 68 h, as proposed in references [52,53] and significant changes in the UV-VIS spectra were not observed (Figure S4). Cell cultures were exposed to different complex concentrations ranging from 20 to 480 µM. Untreated cells were used as controls. The effect of the complex on cell variability was examined at 4 time points. A dose-dependent increase in cytotoxicity was observed at all time intervals (1 h, 4 h, 18 h, and 24 h) as shown in Figure 6. The viability results are presented in Figure S5.

Comparing the IC_50_ values (Figure 6), it is observed that pancreatic carcinoma cells are the most sensitive to the action of the **1** with an IC_50_ of 213.7 µM, 24h. A similar effect was achieved after 18 and 24 h in colorectal adenocarcinoma cell culture (IC_50_: 246.3 µM, 24 h). The cytotoxicity of the **1** is weakest in human breast cancer cells. IC_50_ values (403.9 µM, 24 h) were almost twice as high as in MIA PaCa-2 cells.

This selectivity indicates that various cancer types may display different sensitivities to **1**. The selectivity index ranges from 2 to 6, compared to healthy HS-5 fibroblast-like cells (Table S4). The values are highest for pancreatic carcinoma and colon adenocarcinoma cells, which is consistent with the greatest sensitivity to the cytotoxic effect of the compound. As the previously published results imply, the comparable Ru(II) complexes could efficiently target cancer cells while minimizing adverse effects on healthy tissues, supporting their potential as effective and safe therapeutic options [20].

#### 2.10.2. Malondialdehyde (MDA) and Protein Carbonyl (PC)

**Figure 7:**
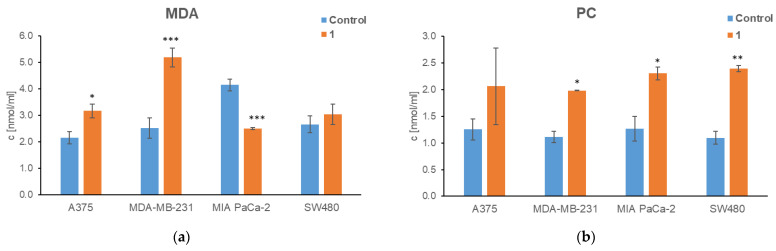
The concentration of (**a**) MDA and (**b**) PC in the medium of untreated and treated cells. * *p* < 0.05; ** *p* < 0.01; *** *p* < 0.01 (comparison to control).

The prooxidative/antioxidant effects of Ru(II) complexes on cancer cells are reflected in the levels of MDA and PC in the medium (Figure 7). Notably, following a 24 h treatment with 1, the concentration of malondialdehyde in MIA PaCa-2 pancreatic cancer cells was significantly decreased (*p* < 0.001). On the contrary, the concentration of PC in supernatant was increased (*p* < 0.05) compared to untreated cells.

The obtained complex has a pronounced oxidant effect on proteins in the medium of all examined cell lines. The concentration of PC significantly increased in MDA-MB-231 (*p* < 0.05) and SW480 (*p* < 0.01) cultures, compared to the control.

Treatment with **1** had the same effects on the amount of lipid oxidation products in the medium. The concentration of MDA was increased in human melanoma (*p* < 0.05) and breast carcinoma cell lines (*p* < 0.001). There was no significant difference in the MDA concentration of the SW480 cell culture.

The findings indicate that **1** has distinct effects on oxidative stress markers in different cell cultures, suggesting other mechanisms of action.

## 3. Materials and Methods

### 3.1. Materials and Instrumentation

Benzophenone hydrazone and [{RuCl_2_(η^6^-*p*-cymene)}_2_], sourced from Tokyo Chemical Industry Co. (TCI, Tokyo, Japan), served as starting materials. 2-propanol and diethyl ether were purchased from Carl Roth. Solvents were dried over suitable molecular sieves for at least two weeks before use to remove residual moisture.

Elemental analysis was performed at the Institute of Chemistry, Chemnitz University of Technology (Heraeus VARIO EL oven). Deuterated solvent, DMSO-*d_6_*, was purchased from Deuterosolvents GmbH and employed in NMR analysis. Proton (^1^H) and carbon (^13^C) NMR spectra, recorded on a Bruker Avance™ 400 MHz spectrometer at 400.23 MHz and 100.1 MHz, respectively, were used to characterize the synthesized complex. FTIR spectrum (ATR technique) was recorded on a Bruker IFS 66v/s FTIR spectrometer.

### 3.2. Synthesis of Dichlorido((diphenylmethylidene)hydrazine-κN)(η^6^-p-cymene)ruthenium(II) Complex

The synthesis was conducted under an inert nitrogen atmosphere utilizing Schlenk techniques to ensure an oxygen-free environment. In a 50 mL flask, [{RuCl_2_(η^6^-*p*-cymene)}_2_] (128.9 mg, 0.2 mmol) and benzophenone hydrazone (103.3 mg, 0.5 mmol) were added and suspended in 2-propanol (25 mL) at room temperature. The mixture was thoroughly stirred and degassed by bubbling nitrogen through a syringe for 15 min. The orange reaction suspension gradually became clear, and a fine precipitate formed immediately. The reaction was allowed to proceed overnight, after which diethyl ether (Et_2_O) was added to the orange suspension. The suspension was then filtered to collect the solid, which was washed three times with diethyl ether (3 × 5 mL) and air-dried. The overall yield was 91.8 mg, corresponding to 45.7%.

Anal. Calcd for C_23_H_26_Cl_2_N_2_Ru (502.44): C, 54.98; H, 5.22; N, 5.58. Found: C, 54,82; H, 5.04; N, 5.52. ^1^H NMR (DMSO-*d_6_*): δ = 7.52 (m, C*H*_Ph_, 2H), 7.44 (m, C*H*_Ph_, 1H), 7.23 (m, C*H*_Ph_, 7H), 5.77 (d, ^3^*J*_H,H_ = 6.2 Hz, CHC*H*_cym_, 4H), 2.79 (h, ^3^*J*_H,H_ = 6.8 Hz, C*H*, 1H), 2.04 (s, CC*H*_3_, 3H), 1.15 (d, ^3^*J*_H,H_ = 7.0 Hz, C(C*H*_3_)_2_, 6H) ppm; ^13^C NMR (DMSO-*d_6_*) δ = 145.37 (N*C*), 139.49 (N*C*_Ph_), 133.76 (N*C*_Ph_), 130.10 (C_Ph_), 129.37 (C_Ph_), 129.28 (C_Ph_), 128.78 (C_Ph_), 127.92 (C_Ph_), 126.12 (C_Ph_), 107.07 (*C*CH(CH_3_)_2_), 100.77 (*C*CH_3_), 87.00 (*C*HCCH_3_), 86.17 (*C*HCCH(CH_3_)), 30.65 (*C*H(CH_3_)_2_), 22.15 (CH(*C*H_3_)_2_), 18.55 (C*C*H_3_) ppm.

### 3.3. Theoretical Calculations

The Gaussian 09 software package [54], employing the B3LYP functional, 6-311++G(d,p) basis set (used for H, C, N, and Cl atoms), and def2-TZVP basis set for Ru(II) ions [55,56,57,58], was used for structure optimization of 1. Confirming the stability of the optimized geometry, the absence of imaginary frequencies indicated that the structure corresponds to a local minimum on the potential energy surface.

For the prediction of NMR spectra and calculation of ^1^H and ^13^C NMR chemical shifts, the structure was reoptimized in the solvent by applying the Conductor-like polarizable Continuum (CPCM) model [59]. The GIAO method [60] was used to determine chemical shieldings, and the chemical shifts were calculated relative to tetramethylsilane (TMS), which served as a reference compound. Special emphasis was put on examining the intramolecular interactions in the structure. The Quantum Atoms in Molecules (QTAIM) approach was used, as described by Bader [61,62]. The Bond Critical Point (BCP) parameters [20] were obtained in the AIMAll program package (Version 19.10.12) [63].

### 3.4. ESR Spectroscopy

#### 3.4.1. ESR Detection of Anti-Hydroxyl Radical (HO^•^) Scavenging Activity

Electron Spin Resonance (ESR) spectroscopy was used to evaluate the ability of compounds to scavenge hydroxyl radicals (HO^•^). Measurements were performed using a Bruker ELEXSYS–II E540 spectrometer (Bruker, Rheinstetten, Germany) operating at X-band frequency (9.51 GHz). The instrument settings were as follows: center magnetic field, 0.3507 T; sweep width, 0.02 T; microwave power, 10 mW; microwave frequency, 9.85 GHz; modulation frequency, 100 kHz; and modulation amplitude, 0.0002 T. The measurements were performed under normal conditions, using quartz capillaries into which Teflon tubes containing 30 µL of sample were placed.

Hydroxyl radicals were generated via a standard Fenton reaction using 5 mM H_2_O_2_ and 5 mM FeSO_4_. The spin trap DEPMPO (5-diethoxyphosphoryl-5-methyl-1-pyrroline N-oxide) [64] was used at a concentration of 0.1 M to trap HO^•^ radicals, following the previously developed protocol [65]. Due to the low solubility of the tested compound in water, it was first dissolved in DMSO to prepare a 1 mM stock solution. The final concentration of the compound in the reaction mixture was adjusted to 16 µM.

Using the abovementioned settings, ESR spectra were recorded 120 s after the reaction began. The scavenging activity of the compound was determined by comparing the average intensity of the two most prominent peaks in the lower field region of the DEPMPO–HO^•^ adduct ESR spectrum. This comparison was made between the control sample (without the test compound) and the sample containing the test compound. The percentage of HO^•^ radical reduction was calculated using the formula:

(1) $$AA=\frac{I_{c}-I_{a}}{I_{C}}100\;(\%)$$

where *I_c_* and *I_a_* refer to the double integral values of the control and sample determined from the EPR spectra (using Xepr software), respectively.

#### 3.4.2. ESR Detection of Anti-Ascorbyl Radical (^•^Asc) Scavenging Activity

The scavenging activity of the samples against ascorbyl radicals (^•^Asc) was assessed using Electron Paramagnetic Resonance (EPR) spectroscopy, following a previously reported method [66]. In brief, a Fe(III)-EDTA complex was prepared by mixing EDTA (final concentration: 250 µM) with FeCl_3_ (final concentration: 8 µM) in DMSO. The test sample, dissolved in a water/DMSO mixture (final concentration: 16 µM), was then added to this solution.

The ^•^Asc radicals were generated by introducing ascorbic acid (final concentration: 250 µM) into the mixture. The reaction solution was then transferred to a gas-permeable Teflon tube for analysis. EPR spectra were recorded 2 min after the addition of ascorbic acid using an X-band spectrometer with the following settings: microwave power of 10 mW, modulation frequency of 100 kHz, microwave frequency of 9.85 GHz, and modulation amplitude of 0.0002 T. Control experiments were conducted by replacing the sample with an equal volume of solvent. The anti-radical activity of each sample was calculated as described previously.

### 3.5. Spectrofluorimetric Determination of HSA Binding Affinity

The binding affinity of 1 to HAS (Sigma, Dorset, UK) and DNA (Sigma, Dorset, UK) was investigated by fluorescence spectroscopy. Measurements were performed on a Cary Eclipse MY2048CH03 spectrophotometer (Agilent Technologies, Santa Clara, CA, USA).

The HSA solution (in PBS, phosphate-buffered saline; pH 7.4) was excited at 285 nm, which is sufficient for activating tryptophan residues. Emission spectra were recorded between 300 and 500 nm with a scan rate of 600 nm min^−1^. Both slits were set to be 5 nm. The concentrations of HSA, NaCl, and KCl (Sigma, Dorset, UK) were 5 μM, 137 mM, and 2.7 mM, respectively. The concentration of 1 was between 1 and 9 μM.

The obtained fluorescence data at different temperatures are further used for Stern–Volmer analysis and assessment of the quenching mechanism. Stern–Volmer (SV) equation has the form:

(2) $$\frac{F_{o}}{F_{Q}}=1+k_{Q}\tau_{Q}\left[Q\right]=1+K_{\mathrm{SV}}\left[Q\right]$$

where F_0_/F_Q_ represents the fluorescence intensity ratio in the absence/presence of quencher molecule; K_SV_—Stern-Volmer quenching constant; k_Q_—quenching rate constant, and τ_0_—fluorophore’s (HSA) lifetime. In this case, there are independent but similar binding sites, and under the assumption of static quenching, the SV equation can be modified into a double-log Stern-Volmer equation, which has the following form:

(3) $$\log\left(\frac{F_{o}-F_{Q}}{F_{Q}}\right)=\log K_{b}+n\;\log\left[Q\right]$$

where K_b_ represents the binding constant and n is the number of binding sites (Hill coefficient) per HSA molecule. Binding constant values at three temperatures, obtained from intercept values on a double-log SV diagram, were used to construct a Van’t Hoff plot and based on that diagram, the values of the Gibbs free energy, enthalpy and entropy of binding were determined following the equations:

(4) $$\ln\;K_{b}=-\frac{∆H_{b}}{\mathrm{RT}}+\frac{{∆S}_{b}}{R}$$

(5) $$∆G_{b}=∆H_{b}-T∆S_{b}$$

### 3.6. Spectrofluorimetric Determination of DNA Binding Affinity

The DNA binding assay was performed by monitoring fluorescence quenching of a complex, using calf thymus DNA (CT-DNA) as the target. The concentration of CT-DNA was determined by measuring absorbance at 260 nm, with a molar absorption coefficient of 6600 dm^3^ mol^−1^ cm^−1^. Experiments were conducted using a Thermo Scientific Evolution 220 spectrophotometer (Thermo Fisher Scientific, Waltham, MA, USA). The concentration of 1 was maintained at 10^−5^ M throughout. The excitation wavelength was set at 217 nm, chosen from the excitation spectrum to achieve optimal fluorescence intensity. Emission spectra were recorded between 237 and 400 nm, with both excitation and emission slits set to 10 nm to accommodate a low fluorescence signal.

A stock solution of CT-DNA (0.474 mM) in phosphate-buffered saline was added incrementally, with fluorescence spectra recorded two minutes after each addition.

Additionally, spectrofluorimetric titrations were performed for the ethidium bromide (EB) displacement studies in the presence of 1. In these experiments, the concentrations of CT-DNA and EB were kept constant at 50 μM and 5 μM, respectively, in phosphate-buffered saline at pH 4 (with NaCl and KCl at 137 mM and 2.7 mM, respectively). The concentration of 1 was varied from 4.74 μM to 42.67 μM in steps of 4.74 μM. The excitation wavelength was set at 520 nm, and emission spectra were recorded between 540 and 650 nm, with both slits set to 10 nm. Thermodynamic parameters related to the displacement process were calculated from the binding constant.

### 3.7. Molecular Docking

Human serum albumin was selected as one of the primary macromolecular targets due to its pivotal role as a carrier protein in systemic circulation. The crystallographic structure of HSA, obtained from the Protein Data Bank (PDB ID: 1H9Z) [67], was utilized for molecular docking studies. As a major transporter of endogenous and exogenous compounds, including pharmaceuticals, HSA is a key player in drug distribution and pharmacokinetics.

In addition to protein targets, polymorphic DNA conformers were also considered, given their potential relevance as binding sites for anticancer drugs. Canonical A- and B-DNA and the non-canonical left-handed Z-DNA represent biologically significant structural forms. To encompass this conformational diversity, four representative DNA structures were retrieved from the Protein Data Bank: the A-form (PDB ID: 3V9D) [68], a B/Z-DNA junction (PDB ID: 2ACJ) [69], and a B/A intermediate structure (PDB ID: 1DC0) [70]. To investigate the process of ethidium bromide displacement from the intercalation site, DNA with (PDB ID: 454D) [71] was used for molecular docking calculations of Ru-complexes in the intercalation site.

Among these, the B- and A-DNA conformers are predominantly observed under physiological conditions, while the Z-form is typically induced under specific environmental conditions or via interactions with certain metal complexes. The Ru(II) complex investigated herein may facilitate localized structural transitions from B-DNA toward A- or Z-DNA [6]. In this context, the Ru(II) complex studied herein may facilitate localized structural transitions from B- to A- and Z-DNA conformations [72]. The calculated Gibbs free energies of binding for each DNA form (A, B, Z, and intermediate) were correlated with experimentally derived Gibbs free energies obtained from fluorescence quenching assays to investigate which DNA form is involved.

All macromolecular targets were pre-processed using BIOVIA Discovery Studio [73], which involved removing co-crystallized ligands, solvent molecules, and redundant polypeptide or nucleotide chains. Only the relevant protein or double-stranded DNA units were retained for docking. The three-dimensional structure of the synthesized complex was optimized using density functional theory (DFT) via Gaussian09 software. The optimization employed the B3LYP functional with the 6-311++G(d,p) basis set for C, H, N, and Cl atoms, and the def2-TZVP basis set for Ru.

Docking input files were prepared using AutoDock Tools v1.5.7, including PDBQT files of macromolecular targets and ligand complexes. Docking simulations were conducted with AutoDock4, using grid maps generated via AutoGrid4. In general docking runs, a grid spacing of 1.0 Å was used to cover the entire molecular surface. For targeted simulations involving confined interaction regions—such as DNA semi-intercalation sites, a finer grid spacing of 0.375 Å was applied, following established protocols.

Each docking study followed the standard AutoDock protocol, comprising ten independent Lamarckian Genetic Algorithm (LGA) runs. For each run, a maximum of 2,500,000 energy evaluations were performed to ensure comprehensive sampling of conformational space and robust identification of energetically favorable binding poses.

### 3.8. In Vitro Activity

#### 3.8.1. Reagents and Chemicals

Dulbecco’s Modified Eagle’s Medium (DMEM), penicillin-streptomycin solution, 0.25% trypsin/EDTA, and phosphate-buffered saline (PBS) were obtained from Gibco. Fetal bovine serum (FBS) was sourced from Capricorn Scientific. MTT reagent (3-(4,5-dimethylthiazol-2-yl)-2,5-diphenyltetrazolium bromide) was purchased from Invitrogen, Thermo Fisher Scientific. Thiobarbituric acid and 1,1,3,3-tetramethoxypropane were obtained from Merck.

#### 3.8.2. Cell Culture and Experimental Design

The human cancer cell lines used in this study included colorectal adenocarcinoma (SW480), breast cancer (MDA-MB-231), pancreatic cancer (MIA PaCa-2), malignant melanoma (A-375), and HS-5 fibroblast-like cells from a healthy donor, all procured from the American Type Culture Collection (ATCC). Cells were cultured in 25 cm^2^ flasks containing DMEM supplemented with 100 U/mL penicillin-streptomycin and 10% FBS and maintained at 37 °C in a humidified atmosphere with 5% CO_2_.

#### 3.8.3. MTT Assay

Cells were harvested upon reaching 85–90% confluence. A volume of 100 µL of cell suspension (containing 15,000 cells per well) was seeded into 96-well plates and incubated overnight until the cells attached to the wells. Cells were then treated with compound 1 in concentrations ranging from 20 to 540 µM, while untreated cells served as controls. The cultures were incubated for 1h, 4h, 18h, and 24 h at 37 °C, before 10 µL of MTT solution (5 mg/mL) was added to each well. The reaction was stopped by adding 100 µL of 10% SDS acidified with 1 M HCl per well. The formazan product was allowed to dissolve overnight at 37 °C. All treatments were performed in triplicate. Absorbance was measured at 540 nm using an ELISA reader (RT-6100, Rayto, Shenzhen, China). The results of the MTT assays are the ratio of treated to untreated cells (positive control). Values from blank wells (medium and MTT reagent, no cells) were subtracted from the values obtained to eliminate background effects [74].

#### 3.8.4. Determination of Malondialdehyde and Protein Carbonyl Levels

Cells (A375, MDA-MB-231, MIA PaCa-2, and SW480) were treated with the IC_50_ concentration of compound 1 for 24 h. Control cells remained untreated. The supernatants were collected for further analysis. Lipid peroxidation was assessed spectrophotometrically by measuring the malondialdehyde (MDA)-thiobarbituric acid complex, which forms under acidic conditions and elevated temperatures. 1,1,3,3-Tetramethoxypropane served as the standard. Absorbance was read at 540 nm using an ELISA reader, and MDA levels were calculated from a standard curve [75]. Protein carbonyl (PC) levels were measured according to the method described by Levine et al., with absorbance readings taken at 375 nm [76].

## 4. Conclusions

A comprehensive investigation of the Ru(II) complex [RuCl_2_(η^6^-*p*-cymene)(bph-κ*N*)] (**1**) was conducted to evaluate its structural, spectroscopic, and biological properties, with an emphasis on its potential anticancer activity. Structural characterization was achieved via FTIR and NMR spectroscopy, with theoretical modeling at the B3LYP/6-311++G(d,p)(H,C,N,Cl)/def2-TZVP(Ru) level showing strong agreement between predicted and experimental bond lengths and angles. QTAIM analysis revealed that the Ru–C and Ru–N bonds exhibit partial covalent character with −G(r)/V(r) ratios around 0.8–0.9 and interaction energies ranging from −130.8 to −92.1 kJ mol^−1^.

EPR-based antioxidant assays revealed that **1** effectively reduced hydroxyl and ascorbyl radicals by 84.6% and 90.1%, respectively, outperforming ascorbic acid. In vitro cytotoxicity assays demonstrated dose- and time-dependent effects, with the lowest IC_50_ observed for MIA PaCa-2 (213.7 µM) and SW480 (246.3 µM) after 24 h exposure. Notably, the complex also influenced oxidative stress biomarkers, significantly increasing protein carbonyl concentrations in multiple cancer cell lines.

Fluorescence quenching studies demonstrated that **1** binds spontaneously to human serum albumin (HSA) and calf thymus DNA (CT-DNA). The thermodynamic analysis yielded ΔG values of −24.3 to −28.6 kJ mol^−1^ for HSA and −21.7 to −26.5 kJ mol^−1^ for DNA, suggesting hydrophobic binding. DNA interaction was further confirmed via EB displacement studies (K_b_ = 1.70 × 10^6^ M^−1^, n = 1.50, ΔG = −35.8 kJ mol^−1^). The specific structure of **1** facilitates its insertion into the target binding site, where it can form the maximum number of π∙∙∙π stacking interactions. Molecular docking results indicate that the change in Gibbs free binding energy becomes more favorable with an increasing number of π∙∙∙π interactions. The most favorable binding energy was observed at the DNA intercalation site (−34.1 kJ mol^−1^), where four π∙∙∙π interactions are established, demonstrating that confined spaces do not hinder binding.

The data highlight **1** as a multifunctional Ru(II)-based agent with promising DNA/HSA affinity, antioxidant potential, and selective cytotoxicity toward MDR cancer cell lines, warranting further pharmacological evaluation.

## Figures and Tables

**Table 1:** The calculated Bond Critical Points (BCPs) properties at the B3LYP/6-311++G(d,p)(H,C,N,Cl)/def2-TZVP(Ru) level of theory of **1**.

Bond	ρ(r) [a.u.]	∇^2^ρ(r) [a.u.]	G(r) [kJ mol^−1^]	V(r) [kJ mol^−1^]	H(r) [kJ mol^−1^]	−G(r)/V(r)	E_bond_ [kJ mol^−1^]
Ru−C1	0.082	0.240	208.9	−260.1	−51.1	0.8	−130.0
Ru−C2	0.083	0.241	209.9	−261.5	−51.7	0.8	−130.8
Ru−C3	0.086	0.239	214.6	−272.7	−58.1	0.8	−136.4
Ru−C4	0.086	0.240	215.0	−272.4	−57.4	0.8	−136.2
Ru−Cl1	0.069	0.184	154.8	−188.8	−34.0	0.8	−94.4
Ru-Cl2	0.067	0.181	151.6	−184.2	−32.6	0.8	−92.1
Ru−N	0.077	0.293	226.0	−259.9	−33.9	0.9	−130.0
Cl1−H	0.008	0.027	14.6	−11.2	3.3	1.3	−5.6
Cl2−H	0.007	0.025	13.4	−10.4	3.1	1.3	−5.2
H−H_p-cym_	0.001	0.005	2.5	−1.6	0.9	1.6	−0.8
H−H_p-cym_	0.002	0.005	2.6	−1.7	0.9	1.5	−0.9

**Table 2:** SV quenching constant, binding constant, the number of binding sites, ∆G_b_, ∆H_b,_ and ∆S_b_.

T [K]	K_SV_ [M^−1^]	K_b_ [M^−1^]	n	∆H_b_ [kJ mol^−1^]	∆S_b_ [J mol^−1^ K^−1^]	∆G_b_ [kJ mol^−1^]	R^2^
300	2.85 × 10^4^	1.65 × 10^4^	0.95			−24.3	
305	3.17 × 10^4^	3.62 × 10^4^	1.01	104.149	482.179	−26.4	0.992
310	3.23 × 10^4^	6.34 × 10^4^	1.06			−28.6	

**Table 3:** The Stern–Volmer quenching constant, binding constant, number of binding sites (Hill coefficient), and thermodynamic parameters of **1** binding to CT-DNA. Fluorescence band at 329 nm.

T [K]	K_SV_ [M^−1^]	K_b_ [M^−1^]	n	∆H_b_ [kJ mol^−1^]	∆S_b_ [J mol^−1^ K^−1^]	∆G_b_ [kJ mol^−1^]	R^2^
300	1.86 × 10^4^	5.34 × 10^3^	0.88			−21.7	
305	1.95 × 10^4^	1.67 × 10^4^	0.98	124.635	487.683	−24.1	0.949
310	1.98 × 10^4^	2.67 × 10^4^	1.03			−26.5	

**Table 4:** Experimentally determined (∆G_exp_) and theoretically calculated (∆G_cal_) change in Gibbs free binding energy values for the **1** bound to HSA, DNA, and a DNA intercalation site. Calculations were conducted for various DNA conformations, including canonical A-DNA and B-DNA, the noncanonical Z-DNA, an intermediate B/A-DNA form, and DNA with a designated intercalation site.

Target	∆G_exp_ [kJ mol^−1^] at 27 °C	∆G_cal_ [kJ mol^−1^] at 25 °C
HSA	−24.3	−23.5
A-DNA	−21.7	−23.0
B/A-DNA		−21.2
B-DNA		−25.1
Z-DNA		−26.8
DNA, intercalation site	−35.8	−34.1

## Data Availability

The original contributions presented in this study are included in the article/Supplementary Materials. Further inquiries can be directed to the corresponding author(s).

## Supplementary Materials

The following supporting information can be downloaded at: https://www.mdpi.com/article/10.3390/molecules30153088/s1, Figure S1: ^1^H and ^13^C NMR of complex **1** (DMSO-d6, 400.23 and 100.1 MHz, respectively); Table S1: Experimental and theoretical (at B3LYP/6-311++G(d,p)(H,C,N,Cl)/def2-TZVP(Ru) level of theory) ^1^H and ^13^C NMR chemical shifts of complex **1**; Figure S2: Fluorescence emission spectra of [RuCl_2_(η^6^-*p*-cymene)(bph-κ*N*)] for the titration with various concentrations of CT-DNA at (a) 27 °C, (b) 32 °C, (c) 37 °C, and (d) Van’t Hoff plot for the binding process (at 329 nm); Figure S3: Fluorescence emission spectra of CT-DNA for the titration with the (a) and the double-log Stern–Volmer dependency of intensity on the concentration of complex **1** (b); Table S2: Structural data of the primary DNA forms; Table S3: Overview of specific interactions between the complex **1** and target biomolecules, including HSA, B/A-form DNA, and DNA containing an intercalation site; Figure S4: Stability examination of complex 1 in DMSO; Figure S5: Viability of A375, MDA-MB-231, MIA PaCa-2, and SW480 cells under the effect of complex **1** for 24 h of treatment (MTT assay); Table S4: The selectivity index for different cell lines; Atomic coordinates of the optimized structure [77,78,79,80].
