# Supplementary material for: Synthesis, Characterization, HSA/DNA Binding, and Cytotoxic Activity of [RuCl2(η6-p-cymene)(bph-κN)] Complex
Source: Molecules. 2025 Jul 23;30(15):3088. doi: 10.3390/molecules30153088 (PMC12348660; doi:10.3390/molecules30153088)
Supplement: Supplementary file 1 [file molecules-30-03088-s001.zip › molecules-3733610-supplementary.pdf]

Synthesis, Characterization, HSA/DNA Binding, and Cytotoxic Activity of [RuCl$_2$(η^6-*p*-cymene)(bph-κ*N*)] Complex

Stefan Perendija [1], Dušan Dimić [1], Thomas Eichhorn [2], Aleksandra Rakić [1], Luciano Saso [3], Đura Nakarada [1], Dragoslava Đikić [4], Teodora Dragojević [4], Jasmina Dimitrić Marković [1,*] and Goran N. Kaluđerović [2,*]

[1] Faculty of Physical Chemistry, University of Belgrade, 11000 Belgrade, Serbia; stefan@ffh.bg.ac.rs (S.P.); ddimic@ffh.bg.ac.rs (D.D.); saska@ffh.bg.ac.rs (A.R.); djura@ffh.bg.ac.rs (Đ.N.)

[2] Department of Engineering and Natural Sciences, University of Applied Sciences Merseburg, D-06217 Merseburg, Germany; thomas.eichhorn@hs-merseburg.de

[3] Department of Physiology and Pharmacology "Vittorio Erspamer", Sapienza University of Rome, 00185 Rome, Italy; luciano.saso@uniroma1.it (L.S.)

[4] Institute for Medical Research, University of Belgrade, 11000 Belgrade, Serbia; dragoslava@imi.bg.ac.rs (D.Đ.); teodora.dragojevic@imi.bg.ac.rs (T.D.)

* Correspondence: markovich@ffh.bg.ac.rs (J.D.M.); goran.kaluderovic@hs-merseburg.de (G.N.K.)

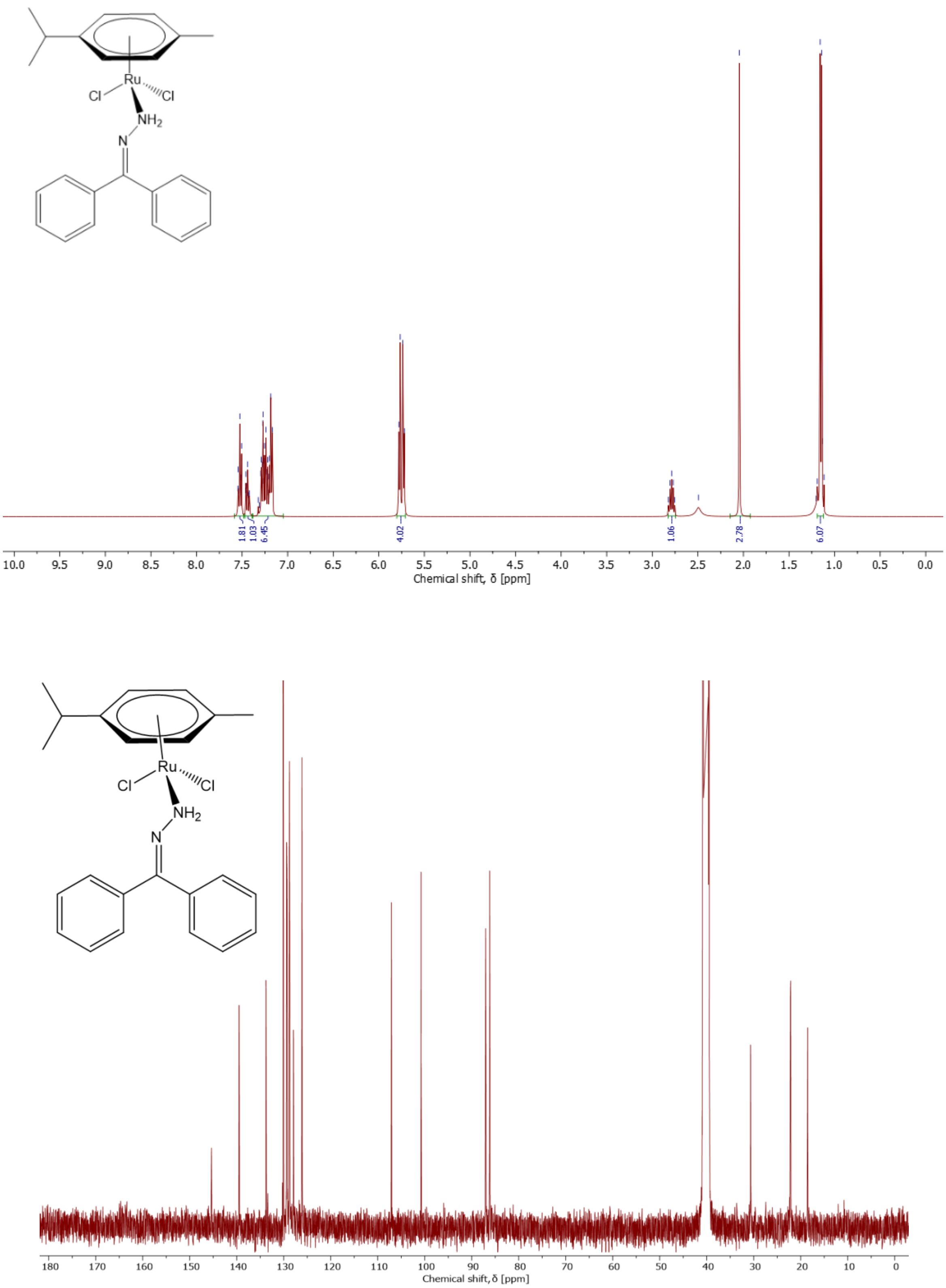

Figure S1. ^{1}H (upper panel) and ^{13}C NMR (lower panel) of **1** (DMSO-d_6, 400 and 100 MHz, respectively)

Table S1. Experimental and theoretical (at B3LYP/6-311++G(d,p)(H,C,N,Cl)/def2-TZVP(Ru) level of theory) ^{1}H and ^{13}C NMR chemical shifts of **1**.

^{1}H			^{13}C		
H atom	Exp. [ppm]	Calc. [ppm]	C atom	Exp. [ppm]	Calc. [ppm]
$C(CH_3)_2$	1.15	1.33	CCH_3	18.55	19.48
CCH_3	2.04	2.01	$CH(CH_3)_2$	22.15	22.56
C*H*	2.79	2.91	$CH(CH_3)_2$	30.65	35.44
$CHCH_{cym}$	5.75	4.66	$CHCCH_3$	87.00	82.21
CH_{Ph}	7.23	7.30	$CHCCH(CH_3)_2$	86.17	81.89
CH_{Ph}	7.44	7.56	CCH_3	100.77	99.07
CH_{Ph}	7.48	8.06	$CCH(CH_3)_2$	107.07	105.29
R		0.992	C_{Ph}	126.12	124.86
MAE [ppm]		0.31	C_{Ph}	127.92	126.66
			C_{Ph}	128.78	127.83
			C_{Ph}	129.28	128.18
			C_{Ph}	129.37	128.62
			C_{Ph}	130.1	129.64
			NC_{Ph}	133.76	129.94
			NC_{Ph}	139.49	133.91
			NC	145.37	166.06
			R		0.997
			MAE [ppm]		3.41

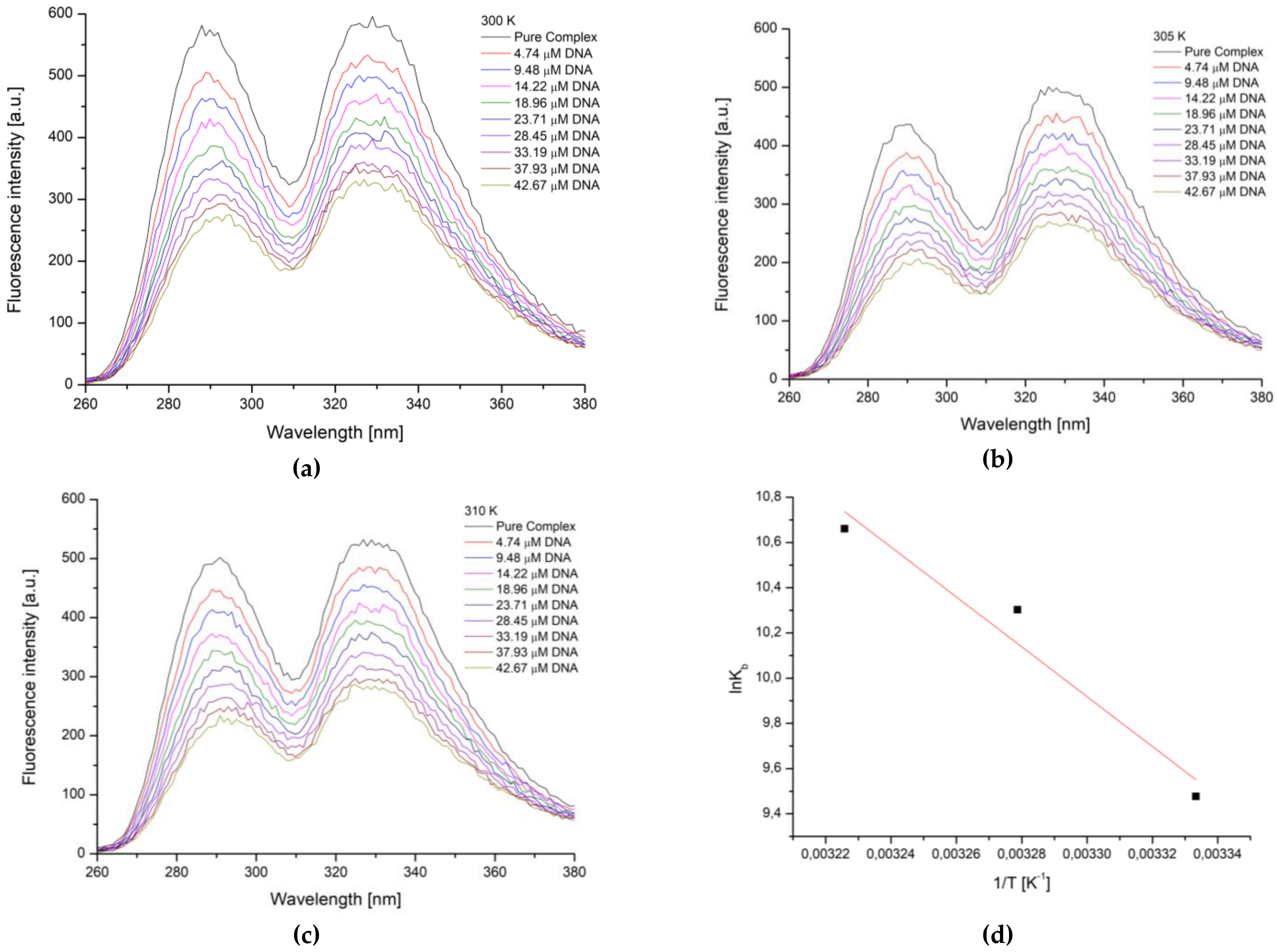

Figure S2. Fluorescence emission spectra of [$RuCl_2$(η^6-*p*-cymene)(bph-κ*N*)] for the titration with various concentrations of CT-DNA at (a) 27°C, (b) 32°C, (c) 37°C, and (d) Van't Hoff plot for the binding process (at 329 nm)

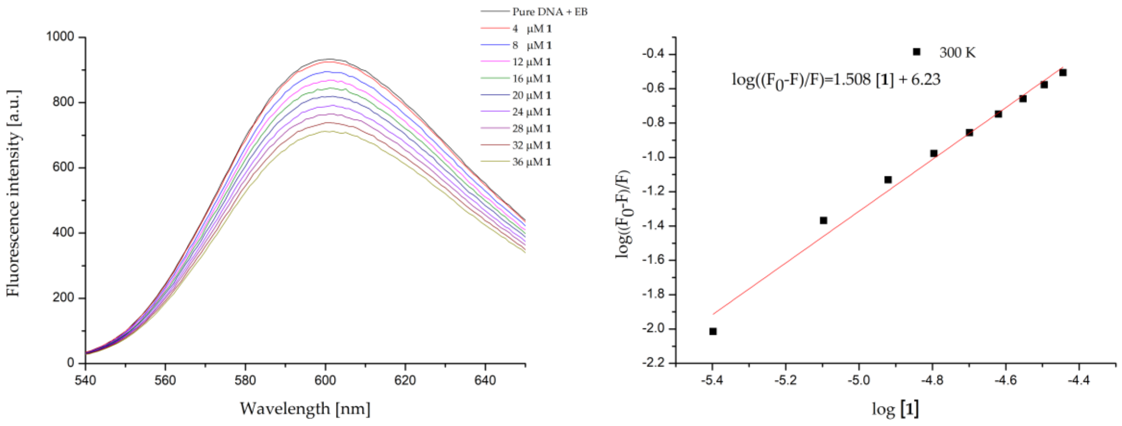

(a) **(b)**

Figure S3. Fluorescence emission spectra of CT-DNA-EB for the titration with the (a) and the double-log Stern–Volmer dependency of intensity on the concentration of **1** (b).

Table S2. Structural data of the main DNA forms.

Feature	B-DNA	A-DNA	Z-DNA
**Helix sense**	Right-handed	Right-handed	Left-handed
**Shape**	Thin, elongated	Shor, broad	Zig-zag backbone
**Base pairs per turn [1]**	10	11	12
**Helix pitch height (Å) [4]**	33.4	30.9 nm	44.6
**Helix diameter (Å) [4]**	20	23	18
**Major groove (Å) [1]**	Width = 11.6<br>Depth = 8.5	Width = 2.2<br>Depth = 13.0	Width = 8.8<br>Depth = 3.7
**Minor groove (Å) [1]**	Width = 6.0<br>Depth = 8.2	Width = 11.1<br>Depth = 2.6	Width = 2.0<br>Depth = 13.8
**Base pair tilt from helix axis**	~6°	~20°	~7°
**Sugar pucker**	C2'-endo	C3'-endo	C2'-endo (pyrimidines), C3'-endo (purines)
**Glycosidic bond conformation**	Anti	Anti	Alternates: purines (syn), pyrimidines (anti)
**Conditions favoring formation**	Physiological (normal)	Dehydrated samples	High salt, negative supercoiling
**Biological occurrence**	Most common in cells	Rare, RNA-DNA hybrids	Regulatory regions, transient state
**Stability**	Very stable	Less stable than B-DNA	Stable under special conditions

The most abundant B-DNA form is stabilized under high humidity conditions (92%) [1] and can be reversibly interconverted into A- and Z-DNA forms. Watson and Crick were the first to describe the B-DNA form as a right-handed double helix with a shallow and narrow minor groove and a deep, wide major groove. The double helix consists of two antiparallel sugar-phosphate backbones with smooth surfaces. Rod-like stacked base pairs in the anti-conformation are connected via hydrogen bonding, linking the two strands of the DNA molecule. The A-DNA form resembles B-DNA but exhibits distinct characteristics: it has a stiffer structure, a deeper and narrower major groove, a shallower and wider minor groove, and a broader, flatter sugar-phosphate backbone. A-DNA occurs less frequently and is often associated with DNA-protein and DNA-RNA complexes. The transition from B-DNA to A-DNA is induced under extreme conditions, such as low water activity, elevated temperature, and acidic pH [1]. The existence of extremophile organisms demonstrates that the A-DNA form plays a crucial role in contributing to survival under extreme environmental conditions [2]. In vitro, A-DNA is stable at low relative humidity (65–75%) and in the presence of metal ions or metal complexes. Substances that precipitate DNA, such as ethanol, can also induce the transition to the A-form. Although standard A and B forms can occur with most DNA sequences, G/C-rich regions are more prone to A-DNA formation [1].

The Z-DNA form does not commonly occur under physiological conditions and differs significantly from A- and B-DNA. It is a left-handed double helix with a zigzag sugar-phosphate backbone. In Z-DNA, purine nucleotides adopt a sin-conformation, while pyrimidine nucleotides are in the anti-conformation. The formation of Z-DNA typically requires an alternating poly (GpC) sequence under high-salt conditions. Moreover, the binding of bulky metal complexes can induce a B-to-Z-DNA transition [1,3].

Table S3. Overview of specific interactions between **1** and target biomolecules, including HSA, B/A-form DNA, and DNA containing an intercalation site.

**1 and HSA (PDB ID: 1H9Z)**		
Ru complex group	Interaction	Amino acid (group)
p-cymene (Benzene-ring)	π···alkyl	Lys195
p-cymene (−CH_3)	alkyl···alkyl	Lys195
p-cymene (−$CH(CH_3)_2$)	alkyl···π	His242 (imidazole-ring)
Benzene1	π···alkyl	Lys195
Benzene1	π···alkyl	Leu198
Benzene1	π···amide stacking	Leu198/Lys199 (amid bond)
Benzene1	π···alkyl	Lys199
Benzene2	π···π, T-shaped	Trp214 (Benzene-ring)
Benzene2	π···π, parallel displaced stacking	His242 (imidazole-ring)
Benzene1	π···alkyl	Lys199
Cl	Cl···H−C carbon hydrogen bond	His242 (imidazole-ring)
**1 and DNA (PDB ID: 1DC0)**		
Ru complex group	Interaction	Nucleotide (group)
p-cymene (−CH_3)	alkyl···π	A:Thy3 (Pyrimidine-ring)
p-cymene (−CH_3)	alkyl···π	A:Ade2 (Pyrimidine-ring)
p-cymene (−CH_3)	alkyl···π	A:Gua24 (Pyrimidine-ring)
p-cymene (−CH_3)	alkyl···π	A:Gua24 (imidazole-ring)
Benzene1	π···π, T-shaped	A:Gua4 (Pyrimidine-ring)
−NH2	N−H···O classic hydrogen bond	A:Gua4 (Sugar-ring)
Cl	Cl···H−C carbon hydrogen bond	A:Thy3 (Pyrimidine-ring)
**Intercalation of 1 in DNA (PDB ID: 454D)**		
Ru complex group	Interaction	Nucleotide (group)
Benzene1-ring	π···π, parallel displaced stacking	A:Gua4 (Imidazole-ring)
Benzene1-ring	π···π, parallel displaced stacking	A:Gua4 (Pyrimidine-ring)
Benzene1-ring	π···π, parallel displaced stacking	A: Cyt5 (Pyrimidine-ring)
Benzene1-ring	π···π, parallel displaced stacking	B: Gua12 (Pyrimidine-ring)
p-cymene (−$CH(CH_3)_2$ group)	alkyl···π	B: Gua12 (Pyrimidine-ring)

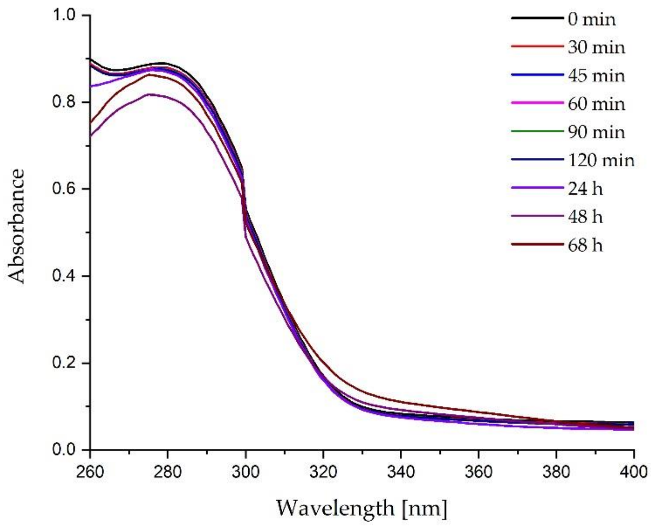

Figure S4. Stability examination of complex 1 in DMSO.

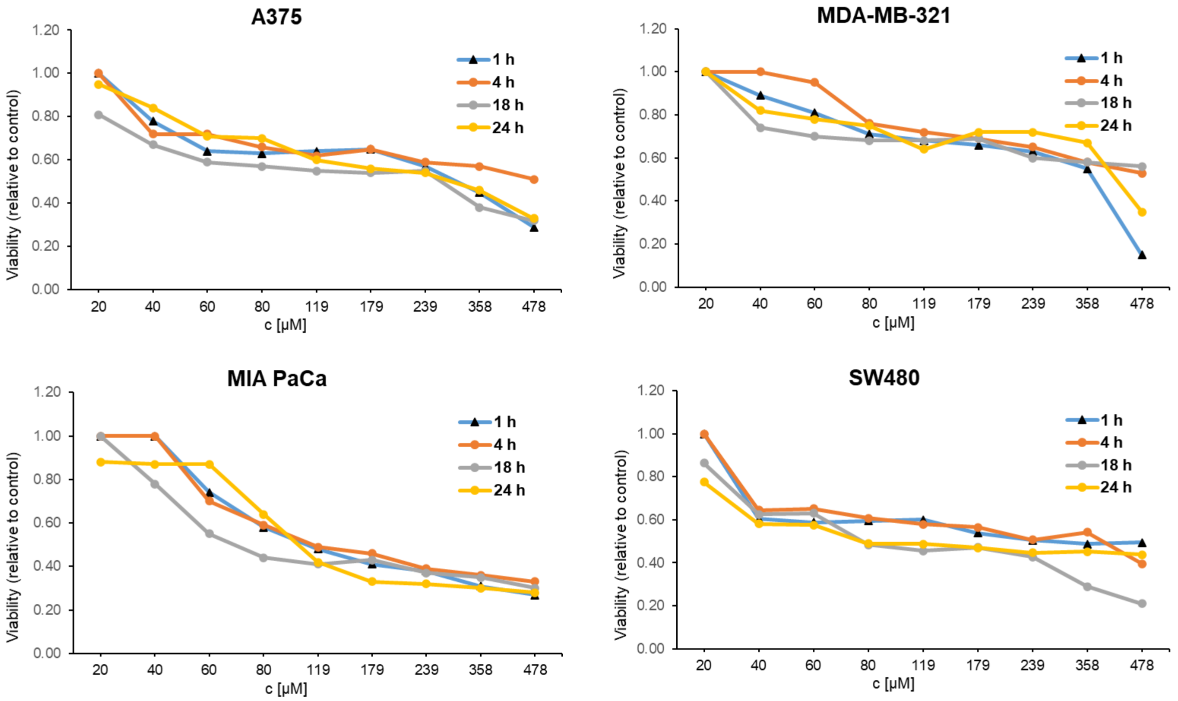

Figure S5. Viability of A375, MDA-MB-231, MIA PaCa-2, and SW480 cells under the effect of **1** for 1, 4, 18 and 24 h of treatment (MTT assay).

Table S4. The selectivity index for different cell lines.

Selectivity Index (SI)				
	**HS-5/A375**	**HS-5/MDA-MB-231**	**HS-5/MIA PaCa-2**	**HS-5/SW480**
**1 h**	3	3	5	2
**4 h**	2	2	5	3
**18 h**	4	2	6	6
**24 h**	3	2	4	4

Atomic coordinates of the optimized structure 1:

No.	Atom	X (Å)	Y (Å)	Z (Å)
1	C	-3.5589070	-0.0441490	0.8124810
2	C	-2.8997240	1.1926640	0.4869310
3	C	-1.5935540	1.3751470	0.9843620
4	C	-0.9665470	0.3852990	1.8088190
5	C	-1.6257110	-0.8114350	2.1550950
6	C	-2.9432020	-1.0169830	1.6190060
7	H	-4.5107980	-0.2692230	0.3481220
8	H	-1.0185440	2.2400070	0.6848600
9	H	0.0588420	0.5365130	2.1238040
10	H	-3.4284050	-1.9740290	1.7629780
11	Ru	-1.6311110	-0.5641140	-0.0651400
12	Cl	-1.0547720	-2.9031900	-0.3655210
13	Cl	-2.5532100	-0.5572530	-2.3215720
14	N	0.2098980	-0.3223960	-1.2274150
15	H	0.6382100	-1.2537410	-1.2377730
16	N	1.0917810	0.7384140	-0.9372110
17	C	-3.6165030	2.2034600	-0.3890800
18	C	-2.6839260	3.1624620	-1.1358360
19	H	-3.2688250	3.7860950	-1.8164180
20	H	-2.1563550	3.8361810	-0.4527110
21	H	-1.9506520	2.6151270	-1.7308360
22	C	-0.9709870	-1.8738810	2.9904070
23	H	-1.4547270	-1.9310970	3.9712320
24	H	-1.0550880	-2.8465910	2.5014350
25	H	0.0876950	-1.6593810	3.1436370
26	C	-4.6482240	2.9747080	0.4595320
27	H	-4.1537510	3.5762180	1.2288700
28	H	-5.2282070	3.6499540	-0.1752720
29	H	-5.3488220	2.3003710	0.9592550

30	H	-4.1556520	1.6207700	-1.1425600
31	H	-0.2269670	-0.1444190	-2.1352060
32	C	2.2667160	0.4617380	-0.4921930
33	C	2.8019790	-0.9318650	-0.3437670
34	C	3.1655770	-1.6674430	-1.4783640
35	C	2.9456530	-1.5133210	0.9206420
36	C	3.6566640	-2.9650550	-1.3486890
37	H	3.0693830	-1.2213240	-2.4624210
38	C	3.4316370	-2.8117640	1.0472890
39	H	2.6775250	-0.9474840	1.8058940
40	C	3.7870840	-3.5399440	-0.0868030
41	H	3.9314610	-3.5262210	-2.2342690
42	H	3.5292930	-3.2566180	2.0309570
43	H	4.1611770	-4.5522790	0.0129180
44	C	3.1439940	1.6102940	-0.1321570
45	C	2.6356750	2.9206690	-0.1107800
46	C	4.4971720	1.4114700	0.1755230
47	C	3.4571050	3.9928170	0.2100520
48	H	1.5944940	3.0807110	-0.3581830
49	C	5.3195860	2.4904260	0.4963730
50	H	4.9127910	0.4120380	0.1586730
51	C	4.8038180	3.7827630	0.5169000
52	H	3.0492840	4.9974540	0.2215680
53	H	6.3643410	2.3171630	0.7281320
54	H	5.4428810	4.6217800	0.7679920

References

1. Neidle S. Principles of Nucleic Acid Structure, 1st ed.; Elsevier: Oxford, UK, 2008; pp. 38–67.
2. Porschke D. Boundary conditions for free A-DNA in solution and the relation of local to global DNA structures at reduced water activity. Eur. Biophys. J. 2016, 45, 413–421.
3. Hangan, A.C.; Oprean, L.S.; Dican, L; Procopciuc, L.M.; Sevastre, B.; Lucaciu, R.L. Metal-Based Drug–DNA Interactions and Analytical Determination Methods. Molecules 2024, 29, 4361.
4. Krall, J.B.; Nichols, P.J.; Henen, M.A.; Vicens, Q.; Vögeli, B.; Structure and Formation of Z-DNA and Z-RNA. Molecules 2023, 28, 843.
